# Turnover of Benzoxazinoids during the Aerobic Deterioration of Maize Silage (*Zea mays*)

**DOI:** 10.1021/acs.jafc.2c06699

**Published:** 2023-01-24

**Authors:** Josef J. Gross, Pierre Mateo, Dietmar Ramhold, Ewald Kramer, Matthias Erb, Christelle A. M. Robert

**Affiliations:** † Veterinary Physiology, Vetsuisse Faculty, University of Bern, 3012 Bern, Switzerland; ‡ Faculty of Science, Institute of Plant Sciences, University of Bern, 3013 Bern, Switzerland; § ISF GmbH, An der Mühlenau 4, 25421 Pinneberg, Germany

**Keywords:** benzoxazinoid, aerobic deterioration, maize, silage, yeasts, moulds

## Abstract

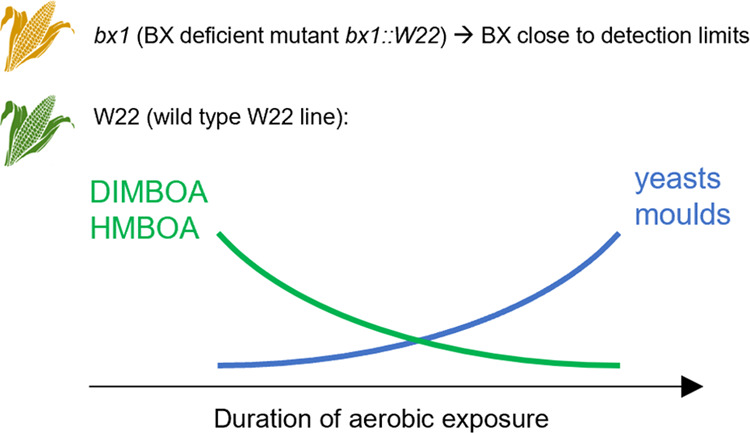

While plant-specialized metabolites can affect mammal health, their fate during the aerobic deterioration of crop silage remains poorly understood. In this study, we investigated the metabolization of benzoxazinoids (BXs) in silages of two maize genotypes (W22 wild type and *bx1* mutant line) during aerobic deterioration. In W22 plants, concentrations of the aglucone BXs DIMBOA and HMBOA in silage decreased over time upon air exposure, while concentrations of MBOA and BOA increased. Mutant plants had low levels of BXs, which did not significantly vary over time. Aerobic stability was BX-dependent, as pH and counts of yeasts and molds were higher in W22 compared to that in *bx1* silage. The nutrient composition was not affected by BXs. These preliminary results may be used to estimate the amounts of BXs provided to farm animals via silage feeding. However, further research is warranted under different harvest and storage conditions.

## Introduction

Benzoxazinoids (BXs) are widespread specialized metabolites in *Poaceae* and are involved in plant nutrition, defenses, and interactions with its environment.^1−4^ Preliminary results of unpublished data indicated that ensiling of chopped maize leads to a decrease in concentrations of BX-glucosides and an increase in the respective benzoxazinone aglucones during the ensiling of maize.^5^ In particular, ensiling anaerobic conditions lead first to the formation of HMBOA and DIMBOA in maize silage and then to the accumulation of MBOA and BOA, stable end products of BX metabolization in silage over 6 months.^5^

Silage enables the preservation of field crops for feeding purposes outside the vegetation period. Lactic acid bacteria are mostly responsible for the fermentation of ensiled crops under anaerobic conditions along with a reduction of the silage pH value.^6^ As long as anaerobic conditions are maintained, the storage period can be extended for several months after harvest. During the feed-off phase (i.e., opening of the silage at feeding), the silage is exposed to oxygen. Consequently, microorganisms and spores that were suppressed by the low pH value and the absence of oxygen (predominantly yeasts and molds) can germinate and proliferate.^7−9^ However, their increase is accompanied by a distinct loss of silage DM and energy reflected by a considerable increase in silage temperature.^9,10^ Furthermore, aerobic deterioration of silage reduces its palatability and may impose threats to animal health and food quality.^11,12^ To date, the fate of plant-specialized metabolites and their impact on the quality of in oxygen-exposed silage remains poorly investigated. After opening, BX in silage can be assumed not to remain stable. The metabolization of BX may not only affect the silage quality but also have consequences for animal performance and health after consumption. Thus, the objectives of the present study were to characterize BX profiles in silage during aerobic exposure and to investigate the possible impact of BX presence on silage quality during aerobic exposure.

## Materials and Methods

### Silage Preparation

Maize (*Zea mays* L.) genotypes of the wild-type W22 line and a near-isogenic line of a BX-deficient *bx1* mutant *bx1::*W22 (referred to as *bx1*, gene identifier GRMZM2G085381; Ds, B.W06.0775)^13^ were cultivated under field conditions (area of 2.53 ha; 10 alternating blocks of 12 rows per genotype, row distance 0.75 m) following conventional Swiss farming practices. Plants were harvested 154 days after seeding and chopped by a self-propelled forage harvester (theoretical chop length: 11 mm).

Immediately after chopping, round silage bales were prepared in a stationary fixed-chamber baler (chamber diameter 1.2 m, width 1.2 m) wrapped with six layers of polyethylene stretch foil (25 μm). Silage bales were stored in a covered shelter with concrete ground. After 26 days of anaerobic fermentation, the silage of the two maize varieties was directly taken from three round bales each at the opening. Replicates (*n* = 3) of samples during aerobic exposure refer to the individual three opened bales per genotype. Forage was loosely and uncompactedly filled into 2 L polyethylene plastic containers that were covered with two layers of laboratory towel to avoid the evaporation of moisture, but to ensure air exchange and oxygen access. Weights of the amount of filled maize and the entire container were recorded. In total, 24 containers (8 scheduled sampling times, 3 replicates each) per genotype were prepared. After filling, containers were stored in a ventilated dark room at 21.4 ± 0.5 °C.

### Data Recording and Sampling

Samples of silages (three replicates per variety and sampling point) were obtained at the filling of the containers (day 0) and on days 1, 2, 3, 5, 7, 10, and 14 of aerobic exposure (8 sampling events in total). Single-use NFC temperature data loggers (ETAG-1, Elitech Technology Inc., Milpitas, California) sealed in a plastic film were used to automatically record temperatures at 15 min intervals. Ambient temperature was measured from the start of silage opening, filling of containers, and throughout the aerobic exposure for 14 days by three data loggers placed randomly in the storage room. Additional four data loggers (three in the containers scheduled for sampling on day 14, one in a container scheduled for sampling on day 10) were placed at filling in the center of the containers (one per container) of each maize variety for recording the silage temperature during the aerobic exposure. The samples were vacuum-sealed and frozen at −80 °C until analysis. Weight loss was determined at all time points by weighing the individual containers.

### Laboratory Analyses

Aerobic stability characteristics were measured in silage sample pools (approximately 500 g of FM from three replicates) per genotype and time point.

The DM content was determined by drying the samples for 48 h in a forced-air oven at 58 °C.^14^ Nutrient composition quality markers were assessed by further milling the dried samples through a 1 mm screen, prior analyses using near-infrared reflectance spectroscopy (NIRS). The NIR-Systems 5000 monochromator (Perstrop Analytical Inc., Silver Spring, Maryland) was used over a wavelength range of 1100–2500 nm in 2 nm intervals. The software NIRS 2 (Infrasoft International, ISI, Port Mathilda, Pennsylvania) was used for scanning, mathematical processing, calibration, validation, and statistical analysis of the spectra data. Silage pH was measured using a standard pH meter (model pH 7310 with pH electrode Sentix 21, Wissenschaftlich-Technische Werkstätten GmbH, Weilheim, Germany, DIN EN 12176) in the laboratory of the ISF Schaumann Forschung GmbH (Wahlstedt, Germany). Fermentation products, i.e., acids (lactic acid, acetic acid, propionic acid, *n*-butyric acid, isovaleric acid) and alcohols (1,2-propanediol, ethanol, *n*-propanol) were analyzed by high-performance liquid chromatography (HPLC) at ISF Schaumann Forschung GmbH (Wahlstedt, Germany). The HPLC system was equipped with a UV detector (Smartline 2500), RI detector (Smartline 2300), column thermostat (model Jetstream 2, all Bio-Rad Laboratories, California), and Aminex HPX-87H-column (300 × 7.8 mm^2^, Bio-Rad Laboratories, California). The mobile phase was sulfuric acid 0.02 N with a flow rate of 0.6 mL/min.

Yeasts and molds were determined on YGC agar (yeast extract glucose chloramphenicol agar; Oxoid, Hampshire, England) according to VDLUFA (III, 2012; method 28.1.2).^15^ Plates were incubated under aerobic conditions at 30 °C for 5 days.

BX concentrations were determined in individual replicates (*n* = 3 per variety) on days 0, 1, 3, and 5 of aerobic exposure using a method adapted from Robert et al.^16^ All forage samples were ground in liquid nitrogen and aliquoted (100 ± 2.5 mg). BX extraction was performed by adding 1 mL of 70:30:0.1 MeOH/H_2_O/FA to the aliquots. All samples were then vortexed (30 s) and centrifuged at 10 °C, 13 Krpm for 20 min. Approximately 750 μL of the supernatant was collected per sample for analysis. BX analysis was performed using an Acquity UHPLC system coupled to a G2-XS QTOF mass spectrometer equipped with an electrospray source (Waters). Gradient elution was performed on an Acquity BEH C18 column (2.1 × 50 mm^2^ i.d., 1.7 mm particle size) at 90–70% A over 3 min, 70–60% A over 1 min, and 40–100% B over 1 min, holding at 100% B for 2.5 min, holding at 90% A for 1.5 min where A = 0.1% formic acid/water and B = 0.1% formic acid/acetonitrile. The flow rate was 0.4 mL/min. The temperature of the column was maintained at 40 °C, and the injection volume was 1 μL. The QTOF MS was operated in positive mode. The data were acquired over an *m*/*z* range of 50–1200 with scans of 0.15 s at a collision energy of 4 V and 0.2 s at a collision energy ramp from 10 to 40 V. The capillary and cone voltages were set to 2 kV and 20 V, respectively. The source temperature was maintained at 140 °C, the desolvation was 400 °C at 1000 L/h, and the cone gas flow was 50 L/h. Accurate mass measurements (<2 ppm) were obtained by infusing a solution of leucin encephalin at 200 ng/mL at a flow rate of 10 mL/min through the Lock Spray probe (Waters).

### Calculations and Statistical Analysis

All data are shown as mean value ± standard error of the mean (SEM). Temperature data from the data loggers were averaged at 8 h intervals. Aerobic stability was defined as the period until silage temperatures exceeded the ambient temperature by more than 2 °C.^17^

Statistical analysis was carried out on SAS, version 9.4; SAS Institute Inc., Cary, NC. M. Generalized linear models were conducted using maize variety and time of aerobic exposure as fixed effects. Replicates within each genotype and sampling point were considered random. Significant effects were declared at *P* < 0.05 using Bonferroni-corrected *t*-tests.

## Results and Discussion

### Limitations of the Present Study Data

It needs to be emphasized that the present data were derived from only one experiment, i.e., a fixed harvest date and one conservation time. The lack of research data does currently not allow drawing generalized conclusions on BX alterations during forage deterioration. This study is the first to investigate changes in BX during the aerobic exposure of maize silage. Nevertheless, changes in plant metabolites during silage storage and the feed-off phase may affect animal productivity and health. Further research including different harvest and storage conditions is warranted.

### Aerobic Stability and Silage Quality

**Figure 1:**
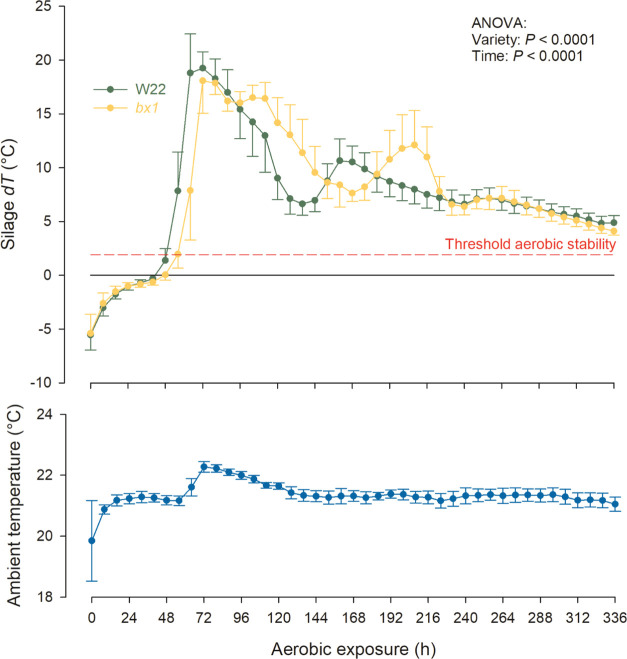
Development of ambient and silage (W22: wild-type W22 line; *bx1*: BX-deficient mutant *bx1::W22*) temperatures during aerobic exposure. Silage temperatures are shown as delta (d*T* in °C) from the ambient temperature. The threshold for aerobic stability was set at 2 °C above ambient temperature. Data are mean values ± SD.

**Figure 2:**
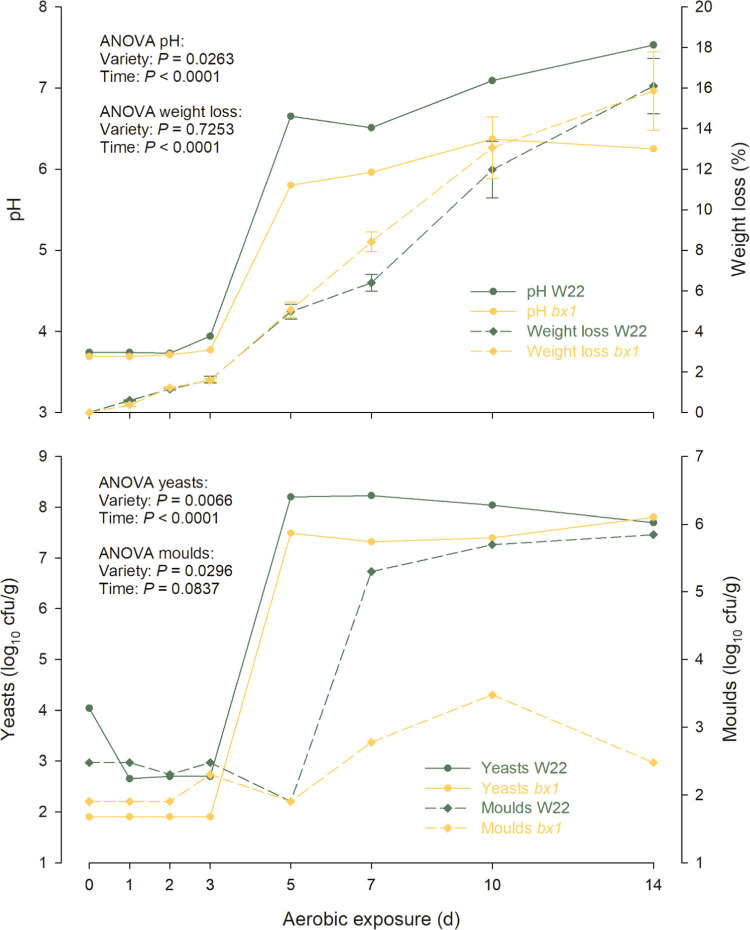
Changes of pH (pooled samples), weight loss (*n* = 3 replicates per variety), and microbial counts (yeasts and molds in pooled samples) in silages of two maize genotypes (W22: wild-type W22 line; *bx1*: BX-deficient bx1 mutant *bx1::W22*).

As a result of aerobic microbial activity, silages deteriorate upon exposure to air.^6,9,18^ The metabolism of lactic and other acids is associated with a rise in the pH and temperature of the silage.^10,17,19^ Ambient temperature for sample storage during the present study was on average 21.4 ± 0.5 °C (Figure 1). Although not different from a practical perspective, aerobic stability was approximately 6 h lower (*P* = 0.0030), and the interval to peak temperature was around 5 h shorter in W22 compared to that in the *bx1* maize (*P* = 0.0301; Figure 1 and Table 1). Peak temperature and the maximum difference between ambient and peak temperature did not differ between the two maize genotypes (*P* > 0.05; Figure 1 and Table 1). Importantly, our findings in terms of silage temperature development show a biphasic curve. Earlier observations associated a first temperature peak to the development of yeasts and aerobic acetic acid bacteria, while a second temperature peak to mold development.^9,20^ Silage pH in the present study increased to a greater extent in W22 compared to that in the *bx1* silage (*P* = 0.0263; Figure 2), whereas weight loss was not related to maize genotypes. Together, this suggests that the presence of BX in the silage leads to the accelerated development of yeasts, bacteria, and mold. Consistently, a greater proliferation of yeasts and molds (expressed as log_10_ cfu/g) was detected in W22 compared to that in *bx1* silage on days 5 and 7 of aerobic exposure (*P* < 0.05; Figure 2). As BXs were previously reported to modulate the root-associated microbiome,^21−24^ it is tempting to speculate that BXs also regulate the microbial communities present in silage. Yet, the specific BX-driven changes in silage microbial communities remain to be investigated.

**Table 1:** Parameters of Aerobic Stability in Maize Silages^a^

	maize variety		
parameter	W22 (*n* = 4)	*bx1* (*n* = 4)	*P*-value
aerobic stability (h)	54.0 ± 1.7	60.6 ± 2.2	0.0030
peak temperature (°C)	44.4 ± 2.2	42.9 ± 0.7	0.2259
interval to peak temperature (h)	70.3 ± 2.4	75.7 ± 3.0	0.0301
maximum difference between ambient and silage temperature (°C)	22.7 ± 2.3	20.8 ± 0.9	0.1772

a Data are mean value ± standard deviation (SD).

The nutritional value of BX-containing silages is thus reduced owing to the loss of fermentation products as well as the formation of microbial toxins.^11,25,26^ This may cause a reduced acceptance and feed intake.^12^ Particularly, bacteria, yeasts, and molds alter silage composition with a resulting loss of dry matter (DM) and nutritional components like WSC, lactic acid, acetic acid, and ethanol that are used as substrates for oxidation and microbial growth.^6,7,9,12,27^ In the initial phase of aerobic deterioration, WSC are rapidly oxidized, whereas more complex constituents such as CP tend to increase.^28^ At the start of the aerobic exposure, the chemical composition and the nutritive value of the two silages in the present study were similar (Table 2). We observed distinct alterations in the composition of fermentation products, which is consistent with literature reports.^6,12^ With the increasing length of deterioration, carbohydrates like starch are degraded.^8,28^ In this regard, yeasts have been widely accepted to be responsible for the onset of silage deterioration.^28^ Yeasts can survive at a fairly low pH and tolerate fermentation products like organic acids better than other aerobic microorganisms under aerobic conditions.^29^ During the 14 days of aerobic exposure, the DM content remained at a higher level (and crude ash content respectively at a lower level) in *bx1* compared to the W22 silage (*P* = 0.0024; Table 2). With the exception of DM and crude fat content, all other nutritive quality figures were significantly affected by the time of aerobic exposure, but not by maize variety (Table 2). The maize genotype was not associated with the changes in fermentation products during aerobic exposure (*P* > 0.05; Table 3). However, in silages of both maize varieties, lactic and acetic acid concentrations declined rapidly within the first days of aerobic exposure (Table 3).

**Table 2:** Changes in Chemical Composition and Nutritive Value of Silages during the Aerobic Exposure (Pooled Samples)^a^

		aerobic exposure (days)								*P*-value	
parameter	variety	0	1	2	3	5	7	10	14	variety	time
dry matter (%)	W22	21.10	21.60	21.43	21.80	21.18	19.51	19.26	17.55	0.0024	0.1111
	*bx1*	22.68	22.70	23.11	22.43	22.60	23.61	23.28	20.40		
crude protein	W22	104.9	102.9	103.0	102.1	97.1	106.8	127.2	138.7	0.5199	<0.0001
	*bx1*	103.9	107.0	105.4	102.5	99.9	99.8	120.1	136.0		
crude fiber	W22	220.9	215.3	215.8	211.1	217.9	243.3	262.1	279.4	0.8028	0.0033
	*bx1*	217.0	218.5	219.9	219.9	235.9	226.3	261.3	257.3		
crude fat	W22	33.3	33.0	32.9	34.1	34.6	32.7	33.3	29.4	0.7551	0.5093
	*bx1*	32.3	33.2	32.3	31.2	34.4	35.0	33.8	33.0		
starch	W22	215.7	230.4	234.0	241.5	257.0	193.4	142.5	95.7	0.4684	0.0030
	*bx1*	221.3	208.9	215.2	218.8	201.0	224.4	136.2	122.3		
WSC	W22	<5.0	<5.0	<5.0	<5.0	<5.0	<5.0	<5.0	<5.0	n.a.	n.a.
	*bx1*	<5.0	<5.0	<5.0	<5.0	<5.0	<5.0	<5.0	<5.0		
NDF_org_	W22	421.5	413.1	412.6	409.2	422.2	458.4	492.8	523.1	0.7405	0.0045
	*bx1*	419.5	422.9	424.3	429.0	460.1	444.1	490.2	485.2		
ADF_org_	W22	237.0	231.6	232.6	224.2	228.2	263.7	309.4	327.6	0.5134	0.0009
	*bx1*	229.9	232.5	233.7	234.7	252.1	244.9	295.3	298.0		
ESOM	W22	584.0	591.6	593.9	592.2	582.9	539.3	446.5	403.0	0.2271	0.0006
	*bx1*	592.3	580.5	585.5	585.3	568.1	576.7	495.6	459.0		
ash	W22	34.7	33.5	36.7	36.9	41.4	46.4	53.1	51.9	0.0273	0.0005
	*bx1*	33.7	35.4	34.6	35.9	36.5	38.5	48.4	47.4		
NEL (MJ/kg DM)	W22	6.28	6.33	6.34	6.37	6.32	6.01	5.57	5.24	0.4996	0.0025
	*bx1*	6.30	6.26	6.26	6.23	6.16	6.25	5.79	5.62		

a Values are expressed in g/kg DM unless stated otherwise.

**Table 3:** Changes in Fermentation Products in Silages during the Aerobic Exposure (Pooled Samples)^a^

		aerobic exposure (days)								*P*-value	
parameter	variety	0	1	2	3	5	7	10	14	variety	time
lactic acid	W22	0.445	0.469	0.459	0.303	0.004	0.006	0.004	0.002	0.1588	<0.0001
	*bx1*	0.503	0.504	0.492	0.444	0.016	0.017	0.007	0.008		
acetic acid	W22	0.076	0.082	0.081	0.055	0.008	0.004	0.006	0.007	0.3011	<0.0001
	*bx1*	0.077	0.082	0.076	0.081	0.023	0.017	0.012	0.012		
propionic acid	W22	<0.001	<0.001	<0.001	0.014	<0.001	<0.001	<0.001	<0.001	0.3506	<0.0001
	*bx1*	<0.001	<0.001	<0.001	0.011	<0.001	<0.001	<0.001	<0.001		
1,2-propanediol	W22	<0.001	<0.001	<0.001	<0.001	<0.001	<0.001	<0.001	<0.001	n.a.	n.a.
	*bx1*	<0.001	<0.001	<0.001	<0.001	<0.001	<0.001	<0.001	<0.001		
ethanol	W22	0.352	0.356	0.334	0.249	0.013	0.016	0.006	0.004	0.0719	<0.0001
	*bx1*	0.445	0.429	0.400	0.384	0.014	0.019	0.005	0.002		
*n*-butyric acid	W22	<0.001	<0.001	<0.001	<0.001	<0.001	<0.001	<0.001	<0.001	n.a.	n.a.
	*bx1*	<0.001	<0.001	<0.001	<0.001	<0.001	<0.001	<0.001	<0.001		
*n*-propanol	W22	<0.001	<0.001	<0.001	<0.001	0.010	0.009	<0.001	<0.001	0.1705	0.5000
	*bx1*	<0.001	<0.001	<0.001	<0.001	<0.001	0.001	<0.001	<0.001		

a Values are expressed in %DM.

### Changes of Benzoxazinoids

**Figure 3:**
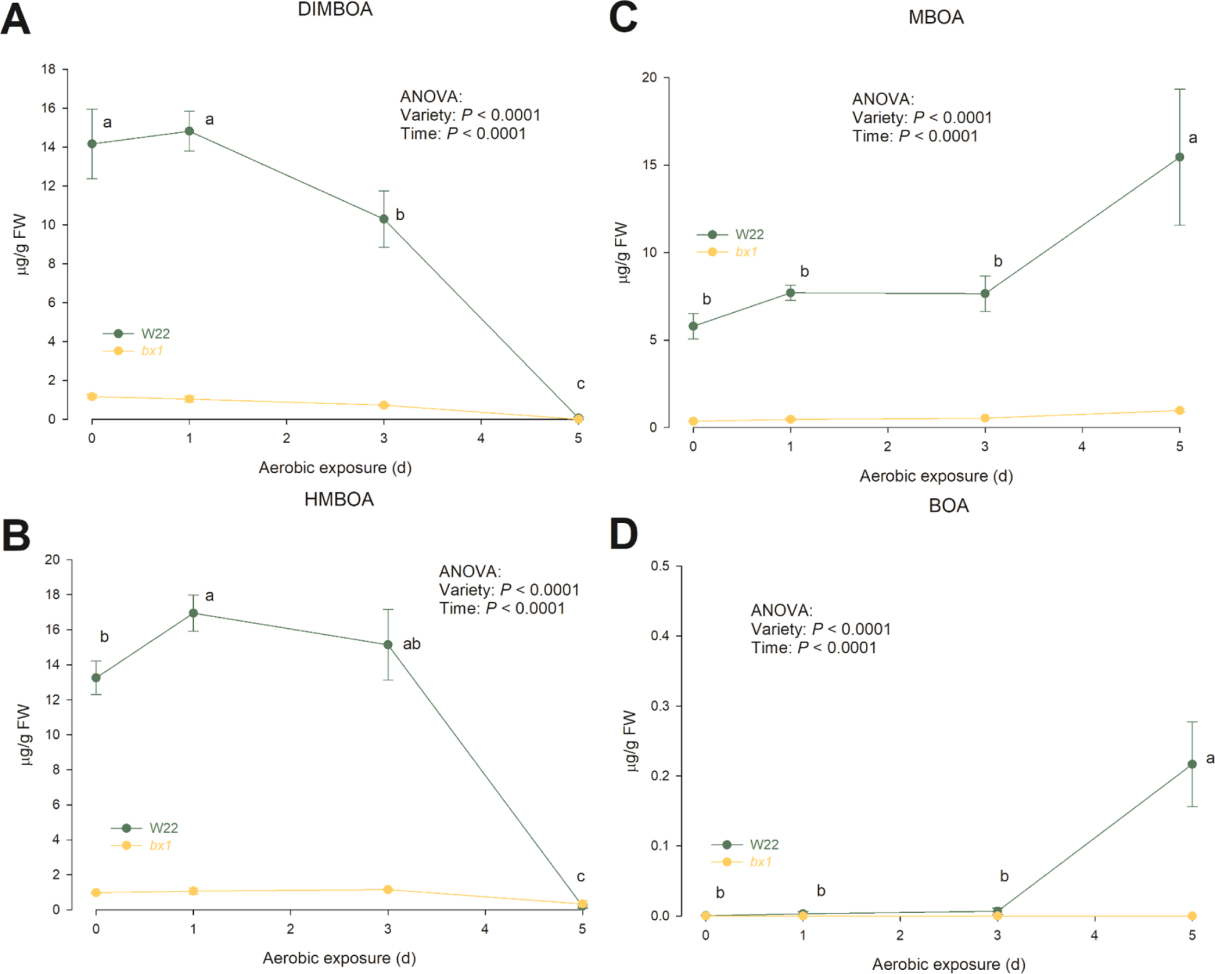
Changes of BX in maize silages of two maize genotypes (W22: wild-type W22 line; *bx1*: BX-deficient bx1 mutant *bx1*::W22; *n* = 3 per variety) during aerobic exposure. Data are mean value ± SEM. Different letters (a, b, c) indicate significant changes over time within the W22 maize silage during aerobic exposure (*P* < 0.05). No changes in BX content were observed over time in the *bx1* maize silage.

As expected, BX concentrations in *bx1* maize silage were at a very low level compared to respective concentrations in the W22 maize (Figure 3). Whereas concentrations of BX did not change during 5 days of aerobic exposure in silage of *bx1* maize, concentrations of DIMBOA and HMBOA in the W22 maize silage started to decline on day 3 and further declined to nadir values close to the detection limit on day 5 of aerobic exposure (Figure 3A,B). In contrast, concentrations of MBOA and BOA were increased on day 5 compared to days 1–3 of aerobic exposure in W22 (*P* < 0.05; Figure 3C,D). In none of the silages, benzoxazinone glucosides (DIMBOA-Glc, DIM_2_BOA-Glc, HMBOA-Glc, HM_2_BOA-Glc, HDMBOA-Glc, HDM_2_BOA-Glc) could be detected, which is characteristic for forages at ensiling.^5^ Only traces of APO and AMPO were found in the W22 silage on day 5 of aerobic exposure, indicating a further degradation of BOA and MBOA, thereby confirming results from BX degradation by bacteria and fungi under aerobic conditions in different environments (e.g., soil, plants, cell culture).^23,30,31^

To conclude, this is the first study depicting BX metabolization in maize silage during aerobic exposure and its impact on silage quality. While DIMBOA and HMBOA concentrations decreased with prolonged aerobic deterioration, concentrations of MBOA and BOA increased in silage produced from a BX-containing maize line. BX further modulated the quality of the silage. In particular, BXs decrease silage aerobic stability, which was supported by a greater pH and counts of yeasts and molds in W22 compared to *bx1* silage. However, results should be interpreted with caution, as further research looking at BX changes at different harvest and storage conditions is necessary. The present results are important for the estimation of bioactive BXs finally consumed by farm animals and may have important consequences for animal health.

## Abbreviations

ADF_org_: acid detergent fiber (exclusive of residual ash)
AMPO: 2-amino-7-methoxyphenoxazin-3-one
APO: 2-aminophenoxazin-3-one
BOA: 1,3-benzoxazol-2-one
BX: benzoxazinoids
bx1: BX-deficient bx1 mutant *bx1::W22* line
CP: crude protein
DIMBOA: 2,4-dihydroxy-7-methoxy-1,4-benzoxazin-3-one
DIM_2_BOA: 2,4-dihydroxy-7,8-dimethoxy-1,4-benzoxazin-3-one
DM: dry matter
ESOM: enzyme soluble organic matter
FM: fresh matter
HDMBOA: 2-hydroxy-4,7-dimethoxy-1,4-benzoxazin-3-one
HDM_2_BOA: 2-hydroxy-4,7,8-trimethoxy-1,4-benzoxazin-3-one
HMBOA: 2-hydroxy-7-methoxy-1,4-benzoxazin-3-one
HM_2_BOA: 2-hydroxy-7,8-dimethoxy-1,4-benzoxazin-3-one
MBOA: 6-methoxy-1,3-benzoxazol-2-one
NEL: net energy lactation
NIRS: near-infrared spectroscopy
NDF_org_: neutral detergent fiber (exclusive of residual ash)
W22: wild-type W22 line
WSC: water-soluble carbohydrates
